# Predictive factors contributing to prolonged recovery in patients after Fontan operation

**DOI:** 10.1186/s12887-022-03537-2

**Published:** 2022-08-24

**Authors:** Yixiao Song, Liping Wang, Mingjie Zhang, Xi Chen, Yachang Pang, Jiaqi Liu, Zhuoming Xu

**Affiliations:** Department of Thoracic and Cardiovascular Surgery, Shanghai Children’s Medical Center, School of Medicine, Shanghai Jiao Tong University, 1678 Dongfang Road, Shanghai, 200127 China

**Keywords:** Children, Fontan, Postoperative, Prolonged recovery

## Abstract

**Background:**

Prolonged recovery is a severe issue in patients after Fontan operation. However, predictive factors related to this issue are not adequately evaluated. The present study aimed to investigate potential predictive factors which can predict Fontan postoperative recovery.

**Methods:**

We retrospectively reviewed the perioperative medical records of patients with Fontan surgery between January 2015 and December 2018, and divided patients with > 75%ile cardiac intensive care unit stay into prolonged recovery group. The others were assigned to standard recovery group. Patients that died or underwent a Fontan takedown were excluded. Statistical analysis was performed to compare data difference of the two groups.

**Results:**

282/307 cases fulfilled the inclusion criteria. Seventy patients were considered in prolonged recovery and 212 patients were defined as standard recovery. Pre- and intra-operative data showed a higher incidence of heterotaxy syndrome, longer cardiopulmonary bypass and aortic cross-clamp time in the prolonged recovery group. Postoperative information analysis displayed that ventilation time, oxygen index after extubation, hemodynamic data, inotropic score (IS), drainage volume, volume resuscitation, pulmonary hypertension (PH) treatment, and surgical reintervention were significantly different between the two groups. Higher IS postoperatively, and PH treatment and higher fluid resuscitation within two days were independent predictive factors for prolonged recovery in our multivariate model. C-statistic model showed a high predictive ability in prolonged recovery by using the three factors.

**Conclusions:**

Ventilation time, higher IS in postoperative day, and PH treatment and higher fluid resuscitation within two days were independent risk factors and have a high predictability for Fontan prolonged recovery.

## Introduction

It was almost half century from the Fontan operation was introduced [1]. As a final stage in the single-ventricle palliation pathway, it completely separates the pulmonary and systemic blood flow, and prevent the mixing of oxygenated and deoxygenated blood. Thereby, it considered to be an important turn point for the treatment of complex cardiac malformation and give patients a chance to survival into adulthood and live with a better quality of life [2, 3].

In recent years, the indication for Fontan operation loosed with the constantly improved surgical methods and perioperative management, which lead to a boarder inclusion of patients for this surgery. Even though the advanced clinical technology and management decreased the incidence of postoperative morbidity and mortality [4, 5], it can’t be ignored that quite a few patients face the issue of prolonged postoperative recovery. And this issues inevitably impact the patient’s prognosis and increase the socioeconomic pressures on their parents. Thus, identifying subtle factors related with prolonged recovery are essential in improving patient’s outcomes.

By exploring the predictive factors, clinicians can quickly recognize the patient’s that may face with obstacles during postoperative recovery, and develop an appropriate effective treatment strategy to improve their outcome. Previous studies illustrated that volume resuscitation, chest drainage, heterotaxia are independent risk factors influencing Fontan recovery [6–8]. However, these factors still need further research to conform and other important potential predictive factors related to prolonged Fontan recovery are still not investigated. In the present study, we aim to make a deep and whole exploration of the predictive factors, including the demographic, perioperative hemodynamic, important procedure and interventions that may contribute to the prolonged recovery on patients after Fontan operation.

## Materials and methods

### Study design

The present study was approved by Health Research Ethics Board of Shanghai Children’s Medical Center affiliated to Shanghai Jiao Tong University School of Medicine. We retrospectively reviewed and collected the clinical data, including the demographic data, preoperative information, surgical method, postoperative hemodynamic and interference, as well as postoperative complications, of patients with Fontan operation from January 2015 to December 2018. We excluded patients who experienced Fontan takedown or didn’t survival to discharge. Patients with > 75%ile cardiac intensive care unit (CICU) stay were defined as prolonged postoperative recovery, and the others were assigned to standard recovery.

### Data collection

Patient’s medical records were reviewed and recorded. The preoperative anthropometric data of weight and height were converted to Z-score by using WHO Child Growth Standards R Macro Calculate to evaluate patient’s nutritional status [9]. Z-score < -2 was defined as malnutrition. Modified Ross classification was used to assess patients heart function before operation. The definition of atrioventricular valve regurgitation requires the regurgitation volume to be above mild to moderate. Mean pulmonary artery pressure (mPAP), mean aorta blood pressure (mABP) and Pp/Ps (mPAP/mABP) in preoperative and immediate postoperative were provided by surgical anesthesia chart. Hemodynamics on postoperative day (POD 0) was obtained by CICU chart and it included both patients on positive pressure ventilation and patients not on positive pressure ventilation. If any drug in treating pulmonary hypertension (PH) was used in POD 0, the mPAP before the treatment was calculated as the mPAP in POD 0.

Specific drugs used to palliative PH were endothelin receptor antagonists and prostacyclin analogues. Resuscitation fluid was defined as the infusion of crystalloid, colloid and blood corpuscle. The volume of resuscitation and chest drainage were standard by patient specific weight to avoid weight-related deviation. The volume of resuscitation and chest drainage and drug therapy in two days after surgery were recorded considering that they may bound with patient’s recovery. Inotropic score (IS) was calculated by using the equation: IS = dopamine (μg/kg/min) + dobutamine (μg/kg/min) + 100 × adrenaline (μg/kg/min) + 100 × noradrenaline (μg/kg/min) + 10 × milrinone (μg/kg/min).

### Statistical analysis

Data analysis was performed by software SPSS 23.0 (IBM, Armonk, NY, USA). Categorical data, normally and non-normally distributed variables were expressed by absolute numbers with percentages, means ± standard deviation and medians with interquartile ranges, and analyzed using x^2^ test, student t-test and Mann–Whitney U-test, respectively. Univariable logistic regression analysis was used to identify the risk factors influencing patient’s outcomes. Multivariable logistic regression model was carried out to determine the independent risk factors associated with prolonged postoperative recovery. In this model, we included factors that had a significant difference between the two groups and may be related with the recovery based on our clinic experience. And we chose forward method to select filter independent variables. Likelihood for the occurrence of prolonged recovery were estimated by using C-statistic model. All statistical tests were 2-sided and considered as significant with a *p* value less than 0.05.

## Results

### Study population

The Fontan operation was performed on total 307 patients from January 2015 through December 2018. Fifteen (including 7 patients diagnosed with heterotaxy) died in the early postoperative and ten (including 2 patients diagnosed with heterotaxy and 1 patient diagnosed with hypoplastic left heart) had Fontan takedown operation patients were excluded in the present study. The remining 282 patients were divided into the two outcome groups based on the time of CICU stay. Seventy patients with the time of CICU stay > 75lie were considered as prolonged recovery, and all of them had a CICU stay > 5d. The other 212 patients with an ICU stay ≤ 75lie/5d were assigned into standard recovery group.

### Preoperative clinical data

The baseline preoperative data of the two groups were displayed in Table 1. Except for the ICU stay (*P* < 0.001) and incidence of heterotaxy (*P* = 0.042), no significant difference was observed in demographic data, diagnosis, modified Ross score, nutrition status, anatomic information, and hemodynamic preoperative data between the two recovery groups. Functional single ventricle includes the primary anatomical deformities of double outlet right ventricle (DVRV), pulmonary atresia (PA), transposition of the great arteries (TGA), tricuspid atresia, Ebstein anomaly, tetralogy of Fallot, hypoplastic left heart and unbalanced ventricle of complete atrioventricular septal defect. DORV was the most common diagnosis up to 35.7% in the prolonged recovery group and 30.2% in the standard recovery group.

**Table 1:** Baseline preoperative data of the two groups

	Prolonged recovery (*N* = 70)	Standard recovery (*N* = 212)	*P* value
ICU stay (day), median (IQR)	7 (6–8)	3 (3–4)	< 0.001^*^
Demographic data			
Sex (male), n (%)	39 (55.7)	126 (59.4)	0.584
Age (year), mean ± SD	4.17 ± 1.789	4.43 ± 1.988	0.317
Height (cm), mean ± SD	100.61 ± 12.957	102.04 ± 12.916	0.423
Weight (kg), mean ± SD	15.65 ± 5.186	16.12 ± 4.709	0.485
BSA (m^2^), mean ± SD	0.66 ± 0.141	0.69 ± 0.135	0.436
Diagnosis, n (%)			0.195
Functional SV	54 (77.1)	178 (84.0)	
SV	16 (22.9)	34 (16.0)	
Modified Ross score, n (%)			0.485
0	58 (82.9%)	182 (86.3%)	
1	12 (17.1%)	29 (13.7%)	
> 1	0 (0.0%)	0 (0.0%)	
Malnutrition, n (%)	16 (22.9%)	46 (21.7%)	0.839
Anatomic information			
Combined with CAVSD, n (%)	11 (15.7)	24 (11.3)	0.334
Combined with TAPVC, n (%)	1 (1.4)	3 (1.4)	1.000
Heterotaxy, n (%)	12 (17.1)	18 (8.5)	0.042^*^
Collateral circulation	97 (45.8%)	37 (52.8%)	0.302
Antegrade flow of Pa, n (%)	21 (30.0)	63 (29.7)	0.964
Common AVV, n (%)	18 (25.7)	33 (15.6)	0.056
Pre-AVVR, n (%)	12 (17.1)	28 (13.2)	0.413
Pre-SpO_2_ (%), median (IQR)	80 (77–85)	80 (80–85)	0.422
McGoon ratio median (IQR)	2.08 (1.7 ~ 2.35)	2.12 (1.79 ~ 2.43)	0.210
Nakata index (mm^2^/m^2^) median (IQR)	249.69 (176.82 ~ 301.48)	244.41 (191.20 ~ 331.30)	0.314
Hemodynamic preoperative data			
mPAP (mmHg), median (IQR)	14 (13–15)	14 (13–16)	0.773
mABP (mmHg), median (IQR)	68 (63–74)	67 (61–74)	0.258
Pp/Ps, median (IQR)	0.21 (0.18–0.24)	0.21 (0.18–0.24)	0.647

*BSA* Body surface area, *SV* Single ventricle, *CAVSD* Complete atrioventricular septal defect, *Pa* Pulmonary artery, *AVV* Atrioventricular valve, *Pre-AVVR* Preoperative atrioventricular valve regurgitation, *Pre-SpO*_*2*_ Preoperative transcutaneous oxygen saturation, *mPAP* mean pulmonary artery pressure, *mABP* mean aorta blood pressure, *Pp/Ps* mean pulmonary artery pressure/ mean aorta blood pressure

^*^ *P* < 0.05

### Operative data

All Fontan operation was performed with cardiopulmonary bypass and 92.9% needed aortic cross-clamp. The most common extracorporeal circulation was full flow (94.0%). And 63.8% operation performed at mild hypothermia and 23.8% at moderate hypothermia. Majority patients needed a fenestration (92.9%). The preferred technique of conduit connection was extracardiac conduit (67.4%). There are 89.5% patients experienced cardioplegia in the extracardiac procedure. Most of them company with other abnormalities (70.0%), including mitral or tricuspid dysfunction, pulmonary stenosis, which required an intracardiac procedure during the Fontan operation. Other conditions such as biventricular repair changed to single ventricular repair also need cardioplegia in the non-intracardiac Fontan procedure. Secondary surgical procedure was mainly pulmonary artery augmentation, systemic atrioventricular valve plasty, atrial septal enlargement. Of all patients, 73.4% underwent stage II Fontan operation. Table 2** s**hows the intraoperative differences between the prolonged and standard recovery groups**.** Statistical analysis showed that only cardiopulmonary bypass time (*P* = 0.004) and aortic cross-clamp time (*P* < 0.001) were factors related with patient’s recovery. There’s no difference in other intraoperative data including the hemodynamics immediately after the surgery.

**Table 2:** Comparison of operative data between prolonged and standard recovery groups

	Prolonged recovery (*N* = 70)	Standard recovery (*N* = 212)	*P* value
CBP time (min), median (IQR)	101 (85 ~ 145)	69 (62 ~ 77)	0.004^*^
Need for aortic cross-clamp, n (%)	66 (94.3%)	196 (92.3%)	0.790
Aortic cross-clamp time (min), median (IQR)	62 (52 ~ 79)	54 (45 ~ 64)	< 0.001^*^
Extracorporeal circulation mode, n (%)			0.645
Low flow	1 (1.4%)	1 (0.5%)	
Parallel circulation	3 (4.3%)	12 (5.7%)	
Full flow	66 (94.3%)	199 (93.9%)	
Temperature, n (%)			0.118
Room temperature	8 (11.4%)	25 (11.8%)	
Mild hypothermia	38 (54.3%)	142 (67.0%)	
Moderate hypothermia	23 (32.9%)	44 (20.8%)	
Deep hypothermia	1 (1.4%)	1 (0.5%)	
Fenestration, n (%)	65 (92.9%)	197 (92.9%)	1.000
Size of fenestration, n (%)			0.329
< 4 cm	3 (4.6%)	8 (4.1%)	
4 cm	56 (86.2%)	180 (91.4%)	
> 4 cm	6 (9.2%)	9 (4.6%)	
Conduit connection, n (%)			0.146
Intracardiac conduits	15 (21.4%)	54 (25.5%)	
Extracardiac conduits	53 (75.7%)	137 (64.7%)	
Intra-extracardiac conduits	2 (2.9%)	21 (9.9%)	
Operation stage (I/II/III), n (%)			0.371
Stage I	7 (10.0%)	12 (5.7%)	
Stage II	48 (68.6%)	159 (75.0%)	
Stage III	15 (21.4%)	41 (19.3%)	
Secondary surgical procedure	44 (62.9%)	136 (64.2%)	0.845
Hemodynamics immediately after surgery			
mPAP (mmHg), median (IQR)	19 (16 ~ 21)	18 (16 ~ 20)	0.267
mABP (mmHg), mean ± SD	56.91 ± 9.824	57.83 ± 7.702	0.428
Pp/Ps, median (IQR)	0.32 (0.27 ~ 0.36)	0.34 (0.27 ~ 0.40)	0.082

*CBP* Cardiopulmonary bypass time, *mPAP* mean pulmonary artery pressure, *mABP* mean aorta blood pressure, *Pp/Ps* mean pulmonary artery pressure/ mean aorta blood pressure

^*^ *P* < 0.05

### Postoperative characteristics

Significant postoperative management associated with prolonged recovery were shown in Table 3. Compare to standard recovery group, prolonged recovery group had longer mechanical ventilation time, and 62.8% patients need more than 24 h mechanical ventilation. Whereas in the standard recovery group, 62.7% patients successfully weaning from the ventilator within 12 h. The oxygen index (P/F ratio) after extubation was significantly higher in patients with a standard recovery. mPAP (*P* = 0.002) and Pp/Ps (*P* < 0.001) were higher and mABP (*P* < 0.001) was lower in POD 0 in the prolonged recovery group when compared with the standard recovery group. Thus, 81.4% patients with a prolonged recovery needed a PH treatment within two days, which only performed in 59.9% patients in the standard recovery group (*P* = 0.034). Endothelin receptor antagonists combined with prostacyclin analogues was the most common method to treat PH. Prolonged recovery patients needed a longer time of chest drainage (*P* < 0.001), and the volume of drainage (*P* < 0.001) and resuscitation (*P* < 0.001) within two days were more than standard recovery group. Otherwise, IS in POD 0 (*P* < 0.001), POD 1 (*P* < 0.001) and POD 2 (*P* < 0.001) was significantly higher in patients with a prolonged recovery. Even IS in POD 2 of the prolonged recovery group was higher than IS in POD 0 of the standard recovery group. Postoperative reintervention mainly included diaphragmatic folding and thoracotomy for hemostasis. Postoperative reintervention (*P* < 0.001), hospital stay (*P* < 0.001) and hospitalization costs (*P* < 0.001) were more in the prolonged recovery, in whom the readmission of early postoperative (*P* = 0.019) was higher. And 74.2% causes of readmission were hydropericardium or hydrothorax. The incidence of hypoxemia (*P* < 0.001), low cardiac output syndrome (*P* < 0.001), liver disfunction (*P* < 0.001) and renal disfunction (*P* < 0.001) were significantly higher in the prolonged recovery group. There’s no significant difference in the incidence of arrhythmia and atrioventricular valve regurgitation between the two groups.

**Table 3:** Postoperative data of the two groups

	Prolonged recovery (*N* = 70)	Standard recovery (*N* = 212)	*P*-value
Mechanical ventilation time (h), median (IQR)	44 (20–95)	10 (8–20)	< 0.001*
Mechanical ventilation time, n (%)			< 0.001*
≤ 12 h	12 (17.1%)	133 (62.7%)	
12–24 h	14 (20.0%)	57 (26.9%)	
24–48 h	15 (21.4%)	16 (7.5%)	
> 48 h	29 (41.4%)	6 (2.8%)	
P/F ratio after extubation, median (IQR)	168.15 (138.64–196.79)	202.06 (165.97–249.03)	< 0.001*
Hemodynamics in POD 0			
mPAP (mmHg), mean ± SD	18.13 ± 3.101	16.80 ± 2.807	0.002*
mABP (mmHg), mean ± SD	58.27 ± 9.095	63.45 ± 9.101	< 0.001*
Pp/Ps, mean ± SD	0.32 ± 0.077	0.27 ± 0.067	< 0.001*
PH treatment after Fontan operation, n (%)	63 (90.0%)	139 (65.6%)	< 0.001*
Drugs in treating PH, n (%)			0.034*
Endothelin receptor antagonists	13 (20.6%)	51 (37.0%)	
Prostacyclin analogues	16 (25.4%)	20 (14.5%)	
Combined	34 (54.0%)	67 (48.6%)	
PH treatment within two days, n (%)	57 (81.4%)	127 (59.9%)	0.001*
Chest drainage time (day), median (IQR)	13 (10–18)	9 (7–14)	< 0.001*
Total drainage volume (ml/kg), median (IQR)	93.12 (62.50–129.56)	41.55 (28.48–61.82)	< 0.001*
Drainage volume within two days (ml/kg), median (IQR)	67.50 (51.25–87.11)	39.38 (27.01–57.89)	< 0.001*
Volume resuscitation within two days (ml/kg), n (%)	132.14 (96.88–185.71)	78.46 (51.72–111.00)	< 0.001*
Inotropic score (IS), median (IQR)			
IS in POD 0 (μg/kg/min)	20.5 (13.5–35)	15 (10.5–18.5)	< 0.001*
IS in POD 1 (μg/kg/min)	20 (13–30)	11 (7.5–14.3)	< 0.001*
IS in POD 2 (μg/kg/min)	17 (10–29.6)	8 (6–12.5)	< 0.001*
Surgical reintervention, n (%)	16 (22.9%)	11 (5.2%)	< 0.001*
Hospital stay (day), median (IQR)	21 (15–28)	15 (12–19)	< 0.001*
Hospitalization costs (dollar), mean ± SD	18,093.81 ± 6953.094	12,535.55 ± 10,039.343	< 0.001*
Readmission within two months after discharge, n (%)	13 (18.6%)	18 (8.5%)	0.019*
Complications			
Hypoxemia, n (%)	49 (70%)	66 (31.1%)	< 0.001*
cardiac output syndrome, n (%)	44 (62.9%)	53 (25.0%)	< 0.001*
Liver disfunction, n (%)	26 (37.1%)	34 (16.0%)	< 0.001*
Arrhythmia n (%)	24 (34.3%)	60 (28.3%)	0.343
disfunction, n (%)	20 (28.6%)	17 (8.0%)	< 0.001*
Atrioventricular valve regurgitation, n (%)	13 (18.6%)	21 (9.9%)	0.054

POD 0 was defined as the time from the patient transfer to CICU to 7 a.m. the next day. POD 1 was the time from 8 a.m. on day 1 of surgery to 7 a.m. the next day. POD 2 was the time from 8 a.m. on the second day of surgery to 7 a.m. on day 3

*P/F ratio* oxygen index, *mPAP* mean pulmonary artery pressure, *mABP* mean aorta blood pressure, *Pp/Ps* mean pulmonary artery pressure/mean aorta blood pressure, *PH* Pulmonary hypertension

^*^ *P* < 0.05

### Prediction factors associated with prolonged recovery time

Univariable and multivariable logistic regression model descripting the relationship between the perioperative data and the recovery were shown in Table 4. Predictors with a significant difference between the two recovery groups were included in our univariable analyses to select the final multivariable model. Considering the confounding between potential factors, we chose two models for the multivariable analysis. In model 1, higher IS in POD 0 (OR 1.031, 95% CI 1.001, 1.061), and PH treatment (OR 2.627, 95% CI 1.053, 6.553) and higher fluid resuscitation (OR 1.015, 95% CI 1.009, 1.021) within two days after the surgery were identified as independent prediction factors for prolonged Fontan recovery. Whereas model 2, mechanical ventilation time (OR 1.051, 95% CI 1.032, 1.071) and fluid resuscitation within two days (OR 1.012, 95% CI 1.006, 1.019) were independent factors in predicting the recovery of Fontan recovery.

**Table 4:** Prediction factors for prolonged recovery after Fontan operation

			Model 1		Model 2	
	Univariable analysis		Multivariable analysis		Multivariable analysis	
	*P*	OR (95%CI)	*P*	OR (95%CI)	*P*	OR (95%CI)
Mechanical ventilation time (h)^a^	0.000	1.056 (1.039–1.073)			0.000	1.051 (1.032–1.071)
Heterotaxy	0.046	2.230 (1.015 ~ 4.899)				
Cardiopulmonary bypass time (min)	< 0.001	1.012 (1.005 ~ 1.018)				
Aortic cross-clamp time (min)	0.002	1.018 (1.006 ~ 1.029)				
Postoperative reintervention, n (%)	< 0.001	5.414 (2.374 ~ 12.346)				
P/F ratio after extubation	0.023	0.995 (0.991 ~ 0.999)				
mPAP in POD 0 (mmHg)	0.002	1.177 (1.060 ~ 1.306)				
mABP in POD 0 (mmHg)	< 0.001	0.939 (0.908 ~ 0.971)				
Pp/Ps in POD 0	< 0.001	9048.856 (135.136 ~ 605,921.471)				
IS in POD 0 (μg/kg/min)	< 0.001	1.050 (1.025 ~ 1.076)	0.042	1.031 (1.001 ~ 1.061)		
PH treatment within two days	0.001	2.935 (1.514 ~ 5.690)	0.038	2.627 (1.053 ~ 6.553)		
Drainage volume within two days (ml/kg)	< 0.001	1.029 (1.018 ~ 1.040)				
Fluid resuscitation within two days (ml/kg)	< 0.001	1.017 (1.011 ~ 1.023)	0.000	1.015 (1.009 ~ 1.021)	0.000	1.012 (1.006–1.019)

*P/F ratio* oxygen index, *mPAP* mean pulmonary artery pressure, *mABP* mean aorta blood pressure, *Pp/Ps* mean pulmonary artery pressure/ mean aorta blood pressure, *IS* Inotropic score, *PH* Pulmonary hypertension

^a^ The factor was not included in the Model 1

### C-statistics model

**Fig. 1:**
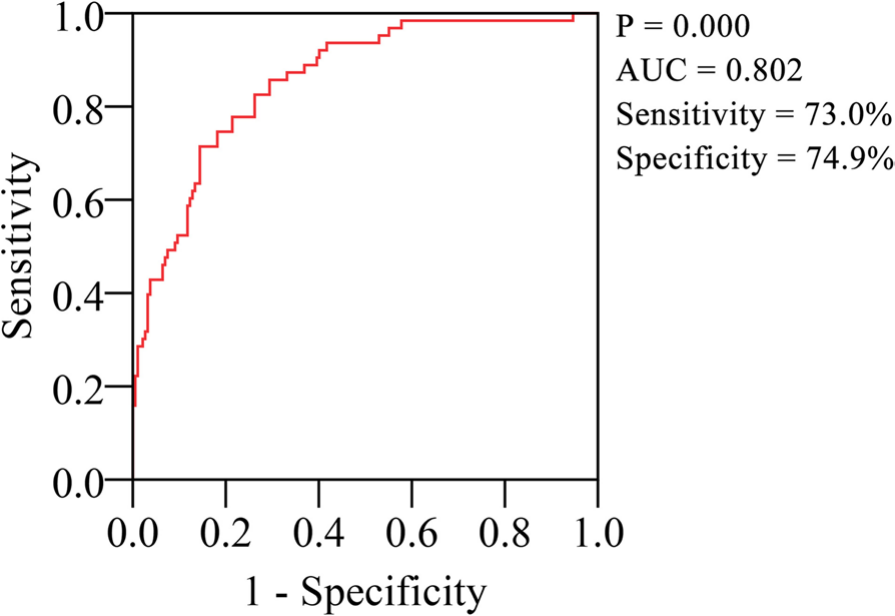
Ability in predicting prolonged Fontan recovery using the three combined factors (IS in POD 0, PH treatment, and fluid resuscitation within two days).

In this part, independent factors in model 2 were input into the prediction model to predict the likelihood of prolonged recovery in patients after Fontan operation due to the potential confounding between ventilation time and other potential factors, such as IS in POD 0, P/F ratio and hemodynamics. Figure 1 reflected the predictive ability of combined factors towards the development of prolonged recovery. The area under the curve of the prediction model was 0.802 (95% confidence interval 0.739 ~ 0.865) with a relatively high sensitivity and specificity. The probability of an individual patient developing prolonged recovery were calculated by the following Eq. [10]:

$$Risk= \pi \left(P\right)=\frac{exp\left(P\right)}{1+exp\left(P\right)}$$

where *P* = -5.036 + 0.03*IS0 + 0.966*PH treatment + 0.015* fluid resuscitation.

## Discussion

Several possible risk factors related to Fontan postoperative complications and recovery were evaluated in recent years [6–8, 11–13]. However, the inconsistency and small sample size nature among previous studies doom the necessity to input more clinical data from different institutions and countries to specify potential variables related to prolonged Fontan recovery. As far as our information goes, the present study was the report with the largest sample size concerning to prediction factors of Fontan recovery in China. Multivariable analysis model showed that ventilation time, higher IS in postoperative day, PH treatment and higher fluid resuscitation within two days after Fontan surgery were independent prediction factors related with prolonged recovery, and C-statistical analysis showed a high predictive ability for prolonged Fontan recovery by using the three combined prediction factors.

### Preoperative

Even though with strict indication for Fontan operation, the early mortality was high at the very beginning. Patients after Fontan palliation benefit from the experience and technical developments of the past few decades and have a significantly improved outcome. Along with the improved surgical methods and perioperative management, the incidence of postoperative mortality decreased to 1% ~ 7% in recent year [14, 15]. In the primary study, the early mortality of Fontan patients was 4.9%.

HLHS is one of the most severe form of congenital heart disease. It was a common primary diagnosis for Fontan operation in most western countries [16, 17]. However, HLHS is a relatedly rare disease in China. The severity of this disease lead to some patients abandoned by parents or died before they could get effective medical assistance. Thus, only two patients were diagnosed as HLHS in the present study, and one had Fontan take-down and one experienced prolonged postoperative recovery. Some studies found that heterotaxy was a risk factor for haemodynamic instability and poor prognosis in Fontan patients [18, 19]. During this work, the incidence of heterotaxy in prolonged recovery group was higher than that in standard recovery group (17.1% vs 8.5%, *P* = 0.042). However, heterotaxy didn’t contribute to prolonged recovery in our multivariable model. It may result from the exclude standard of this study, which due to 9 cases, account for 23% of all patients diagnosed with heterotaxy, counted out in this study.

Several reports declaimed that atrioventricular valve regurgitation (AVVR) is associated with the adverse events in patients after Fontan palliation and fast the procedure of Fontan failure, and up to 20% patients have a moderate or severe AVVR [20, 21]. In the present study, the incidents of AVVR above mild to moderate before (14.2%) and after (12.1%) Fontan operation was very low, which may benefit short-term and long-term recovery.

### Intraoperative

Intraoperative data of longer bypass and aortic cross-clamp time, no fenestration, Fontan stage and instability hemodynamics have described as risk factors for Fontan failure in previous studies [22–24]. Similar analysis was performed in the present study, even though longer bypass and aortic cross-clamp time were found associated with Fontan recovery in univariable analysis, none of them contribute to prolonged recovery in multivariable model. In many prior reports, the fenestration in patients has been associated with hemodynamic stability [25, 26]. Therefore, 92.9% Fontan surgery in our institution were performed with a fenestration to palliate the PAP. Even though hemodynamic information of mPAP, mABP and Pp/Ps in the immediate postoperative were similar between the two groups, it was obviously increased when compared with preoperative hemodynamics.

### Postoperative

PH was also found to be key contributor to both long ICU stay and early death after Fontan operation [23, 24]. It appears logical that increased pulmonary vascular pressure and resistance may contribute to prolonged chest drainage and incubation and reduced exercise tolerance. However, these factors were confounding problem contribution to postoperative morbidity and hospitalization. Spontaneous breathing is important for hemodynamic stability and recovery [27, 28]. Thus, the idea of early extubation is well-accepted and performed in our institution. Benefit of early extubation and PH treatment, mPAP and Pp/Ps of the standard recovery group were decreased to 13 mmHg and 0.27 in the postoperative day from 18 mmHg and 0.34 in the immediate postoperative respectively in the present study. Whereas, hemodynamics of the prolonged recovery group didn’t get any improvement and kept in high level in the postoperative day. Otherwise, the mechanical ventilation time and incidence of hypoxemia was higher, and P/F ratio after extubation was lower in the prolonged recovery group when compared with the standard recovery group in our univariable analysis. It implies that patients were experiencing troublesome recovery.

Salvin [7] et.al demonstrated that a larger fluid resuscitation was the only independent factor for predicting prolonged postoperative recovery after the Fontan surgery in a report of 2008. Sasaki [8] et.al evaluated pre- and intraoperative data and found that worse hypoxemia and increased pulmonary artery pressure were associated with prolonged hospital stay. However, crucial postoperative clinical data, including P/F ratio after extubation, IS, PH management and chest drainage, which may associate with Fontan recovery were not provide by these studies. In our multivariate model, higher IS in POD 0, PH treatment and higher fluid resuscitation within two days after Fontan surgery all were independent prediction factors associated with prolonged Fontan recovery. C-statistic model showed a high area under the curve, sensitivity, and specificity in predicting the probability of prolonged recovery by using the three combined factors.

IS has been proposed as a predictor of cardiac function and fluid balance in clinical. It was found that higher IS were associated with poorer outcomes in patients after cardiac surgery [29, 30]. Early PH treatment is a confounding factor. On the one hand, it indicates that patients are under an unstable hemodynamic status. On the other hand, it is an effective way to decrease the preload, decrease the incidence of adverse event and improve outcomes. Volume expansion is essential in the early postoperative period, which helps maintain adequate preload and cardiac output. Higher IS in POD 0, PH treatment and higher volume resuscitation within two days after Fontan operation potentially imply unstable hemodynamic status and cardiac stress, which in turn increases the hospitalization costs and readmission early after the operation and the incidence of LOCS and organ disfunction.

Inevitably, there were some limitations in this present study. One of the major limitations was the retrospective nature and physician subjected clinical management. However, the present study provided an overall review of related factors and all factors spread randomly across these two groups. Secondly, this study is limited by its single-center design. Nevertheless, it still can represent the status of Fontan postoperative recovery because most of the patients came from different regions of China, not just from Shanghai. Thirdly, some variables, such as ventricular function and end-diastolic pressure, pressure and resistance in the pulmonary arteries and the vessels and cavities of the heart were missing for the reason that only few patients took the cardiac magnetic resonance examination or catheterization during the therapy. We are still collecting relevant data and hope to address this limitation in future research.

## Conclusions

In the present study, we expressed our concern about risk factors affecting normal postoperative recovery in patients after Fontan operation, and hope to provide important information to help physician recognize patients that will experience a longtime recovery. Multivariate analysis showed that ventilation time, higher IS in POD 0, PH treatment and higher fluid resuscitation within two days after Fontan operation were independent risk factors related to prolonged postoperative recovery. C-statistic analysis displayed a high predictive ability in prolonged recovery by using the three combined risk factors.

## Data Availability

The data set supporting the results of this article are included within the article.

## Abbreviations

IS: Inotropic score
PH: Pulmonary hypertension
CICU: Cardiac intensive care unit
mPAP: Mean pulmonary artery pressure
mABP: Mean aorta blood pressure
POD 0: Postoperative day
DORV: Double outlet right ventricle
SV: Single ventricle
PA: Pulmonary atresia
TGA: Transposition of the great arteries
HLHS: Hypoplastic left heart
AUC: Area under the curve
AVVR: Atrioventricular valve regurgitation
